# Pharmaceutical Equivalence of Distributed Generic Antiretroviral (ARV) in Asian Settings: The Cross-Sectional Surveillance Study – PEDA Study

**DOI:** 10.1371/journal.pone.0157039

**Published:** 2016-06-20

**Authors:** Vorapot Sapsirisavat, Vorasit Vongsutilers, Narukjaporn Thammajaruk, Kanitta Pussadee, Prakit Riyaten, Stephen Kerr, Anchalee Avihingsanon, Praphan Phanuphak, Kiat Ruxrungtham, PEDA study team

**Affiliations:** 1 HIV-NAT, Thai Red Cross AIDS Research Centre, 104 Ratchadamri Road, Pathumwan, Bangkok, 10330, Thailand; 2 Faculty of Pharmaceutical Sciences, Chulalongkorn University, Rama 4 Road, Pathumwan, Bangkok, 10330, Thailand; 3 Department of Global Health, Academic Medical Center, University of Amsterdam, Amsterdam Institute of Global Health and Development, Trinity Building C, Pietersbergweg 17, 1105 BM, Amsterdam Zuidoost, The Netherlands; 4 Department of Medicine, Faculty of Medicine, Chulalongkorn University, 254 Phyathai Road, Pathumwan, Bangkok, Thailand; Centro de Biología Molecular Severo Ochoa (CSIC-UAM), SPAIN

## Abstract

**Objectives:**

Ensuring that medicines meet quality standards is mandatory for ensuring safety and efficacy. There have been occasional reports of substandard generic medicines, especially in resource-limiting settings where policies to control quality may be less rigorous. As HIV treatment in Thailand depends mostly on affordable generic antiretrovirals (ARV), we performed quality assurance testing of several generic ARV available from different sources in Thailand and a source from Vietnam.

**Methods:**

We sampled Tenofovir 300mg, Efavirenz 600mg and Lopinavir/ritonavir 200/50mg from 10 primary hospitals randomly selected from those participating in the National AIDS Program, 2 non-government organization ARV clinics, and 3 private drug stores. Quality of ARV was analyzed by blinded investigators at the Faculty of Pharmaceutical Science, Chulalongkorn University. The analysis included an identification test for drug molecules, a chemical composition assay to quantitate the active ingredients, a uniformity of mass test and a dissolution test to assess in-vitro drug release. Comparisons were made against the standards described in the WHO international pharmacopeia.

**Results:**

A total of 42 batches of ARV from 15 sources were sampled from January–March 2015. Among those generics, 23, 17, 1, and 1 were Thai-made, Indian-made, Vietnamese-made and Chinese-made, respectively. All sampled products, regardless of manufacturers or sources, met the International Pharmacopeia standards for composition assay, mass uniformity and dissolution. Although local regulations restrict ARV supply to hospitals and clinics, samples of ARV could be bought from private drug stores even without formal prescription.

**Conclusion:**

Sampled generic ARVs distributed within Thailand and 1 Vietnamese pharmacy showed consistent quality. However some products were illegally supplied without prescription, highlighting the importance of dispensing ARV for treatment or prevention in facilities where continuity along the HIV treatment and care cascade is available.

## Introduction

In 2014, there were an estimated of 450,000 people living with HIV in Thailand, with 8000 total new infections[1]. Since there is no definitive cure, HIV is a chronic disease requiring life-long treatment. In October 2014, new Thai National HIV/AIDS guidelines were launched which recommend initiating treatment “regardless of CD4 count”[2]. Early ARV initiation has public health benefits by minimizing sexual transmission of HIV[3], and also benefits the individual by preventing the development of serious AIDS and non-AIDS-related events[4]. While there is an attempt to scale up ARV for HIV infected individuals, drug accessibility remains a global challenge, especially where financial resources are constrained[5]. In Thailand, 100% of financial resources for HIV treatment are domestic[1]; the country depends mostly on generic ARV products.

Generic drugs, according to U.S. food and drug administration (FDA), are the same as their branded counterparts in dosage, strength, safety, route of administration, indication and action; in fact, they are supposed to be therapeutically equivalent. By FDA regulations, a generic drug must contain an identical amount of the active ingredient(s) as in the branded product[6]. The active ingredient is any component in a tablet that produces the pharmacological effect for an expected medical purpose[7]. However, an active ingredient is just one component of the quality requirements, and is not in itself sufficient to ensure therapeutic equivalence. The Thai FDA has guidelines for approval of generic drugs which requires evidence of product interchangeability equivalence, namely bioequivalence studies, comparative in vitro dissolution/release studies, comparative clinical studies, and comparative pharmacodynamics studies[8]. The World Health Organization (WHO) has a recommended survey protocol that defines specific items that relate to quality aspects of medicines, and substandard and counterfeit medicines. Substandard medicines are legal products that do not meet quality standards and specifications; they may occur as a result of human error, negligence, or resource restriction. In contrast, counterfeit or fake drugs are intentionally and fraudulently disguised regarding drug components, active ingredients, packaging and labeling, and are made illegally by non-licensed companies[9].

Incidents regarding poor-quality generic drugs have been regularly reported, particularly among life-saving anti-infective drugs within resource-limiting regions where there are less rigorous restrictions on procurement and sale, and less public awareness[10–12]. According to the U.S. Pharmacopeial Convention (USP) reports during 2003–2013, the proportion of substandard medicines in Asia was 2.9%; lower than that described in Africa and South America. However, Asia was reported to have the highest proportion of counterfeit medicines with a total number of 70 samples (out of 81 counterfeit products), representing 86% of sampled counterfeit products. Prevalence of sampled substandard medicine in Asia was 2.9% and Thailand was one of the least consistent in reporting data to a medicines quality database[13]. Problems associated with poor-quality drugs include increased morbidity and mortality, unnecessary adverse effects, suboptimal treatment leading to drug resistance, and also loss of confidence in health systems and waste of financial resources[14]. Therefore, it is very important for generic ARVs to be consistently monitored. Our study sampled generic ARV available from different sources in Thailand and assessed the quality by analyzing the pharmaceutical equivalence of the products.

## Materials and Methods

The methodology has been reported in accordance with existing literature on medicines’ quality surveys[9,15].

### Sampling from sources

**Fig 1:**
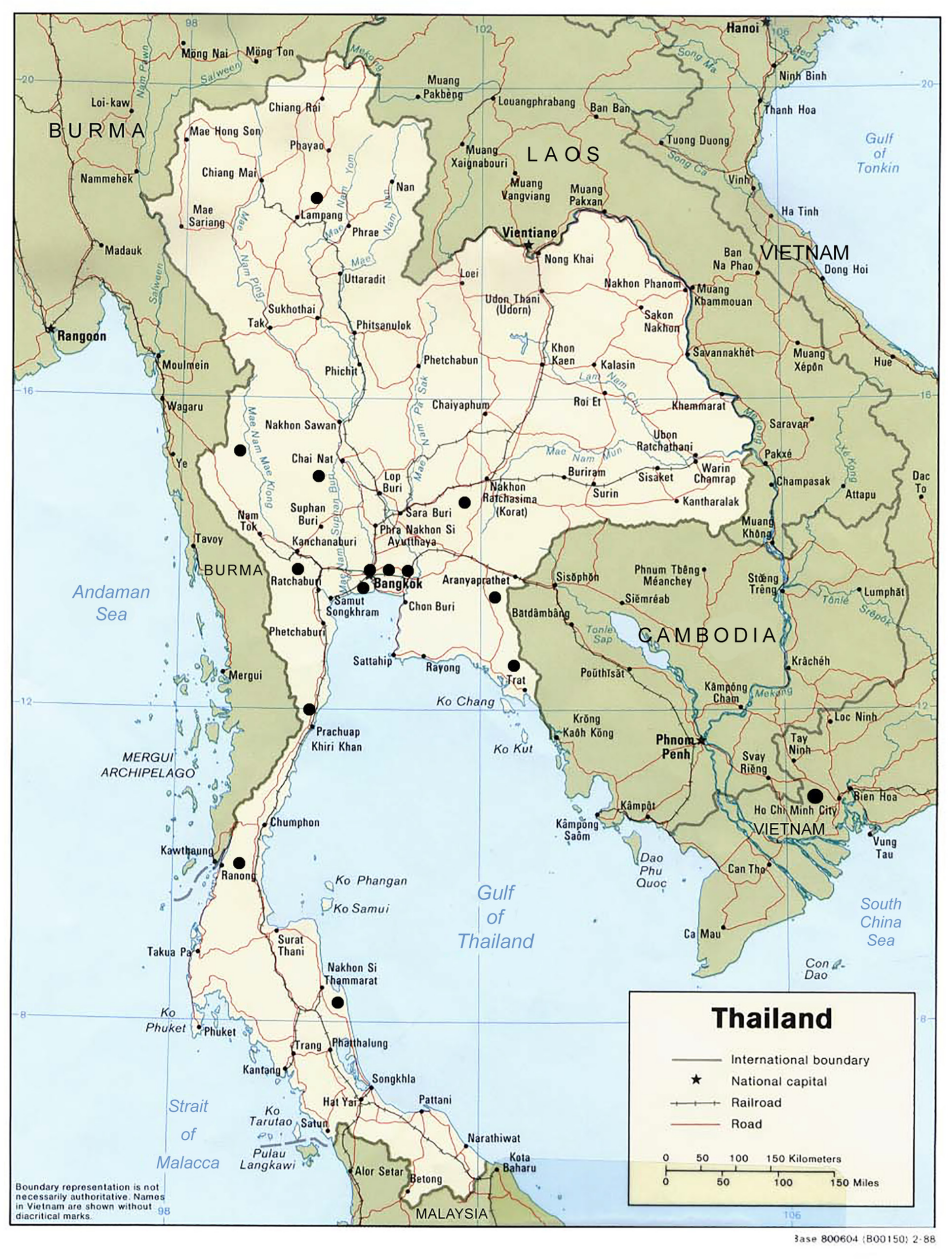
Geographic location of sampling sites in Thailand and Vietnam.

In order to represent ARVs distributed in Thailand, we obtained ARVs from primary hospitals participating in the National Universal Coverage program under the auspices of the National Health Security Office (NHSO), non-government organization (NGO) ARV clinics, and private drug stores. Six hundred and three primary hospitals that distributed ARVs through the NHSO (assessed data August 2011) were grouped by geographical location (North, South, East, West, and Central), and 2 hospitals from each region were randomly selected. For NGO sources, we collected ARV from the Thai Red Cross AIDS Research Center and the HIV-Netherlands-Australia-Thailand Research Collaboration (HIVNAT) pharmacies. For private sources, we bought ARV from 2 private pharmacies in Bangkok and 1 private pharmacy in Vietnam. [Fig 1] Convenience sampling was used to select these ARV in locations where the HIV prevalence is high.

### Selection of ARVs

**Fig 2:**
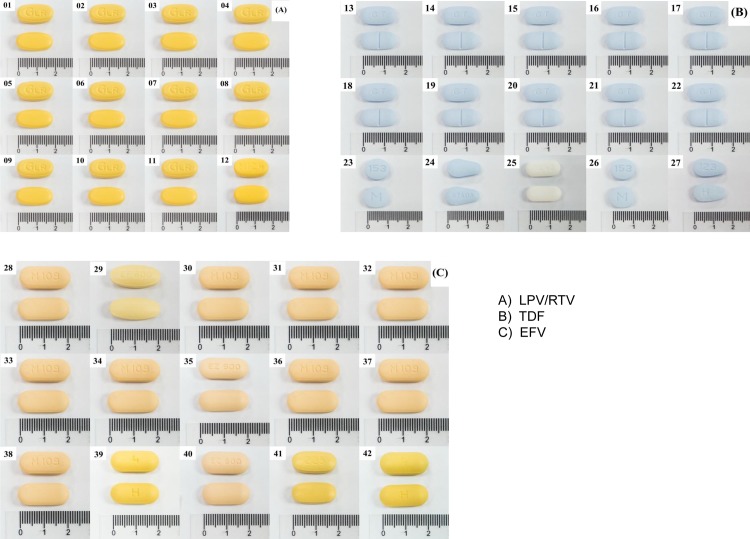
Sampled drugs, ordered by site ID.

We selected two preferred first line ARVs (Tenofovir (TDF) 300mg and Efavirenz (EFV) 600mg) and one preferred second line ARV (Lopinavir/ritonavir (LPV/r) 200/50mg) recommended in the Thai National HIV/AIDS guidelines for this study[2]. Each ARV sample contained an adequate amount of tablets for Pharmaceutical analysis (at least 90 tablets), and had a shelf life extending beyond the analysis date. [Fig 2]

### Collection of ARVs

For hospital sources, Local Pharmacists (who voluntarily collaborated with this study) were asked to randomly collect 1–3 bottles of each designated ARV from dispensing shelves. In addition, they completed a drug record form for each ARV sample, recording information including product name, dose, batch number, storage condition, manufactured date and expiry date. Pharmacists then shipped sampled ARVs along with completed drug record forms to the study team. Transportation to the analysis site was done using the Thai Express Mail Service and temperature was monitored during shipment.

For NGO ARV clinics and private sources, ARVs were bought by study coordinators. They acted as mystery shoppers and did not declare the objective of study to the seller. LPV/r (200/50mg) tablets were unavailable at 2 NGO clinics and 1 private pharmacy. Required documents including temperature assessment were not available for ARV purchased from the private pharmacies.

### Pharmaceutical Analysis of ARVs

Evaluation of ARVs quality was conducted according to standard procedures for pharmaceutical analysis described under specific product monographs, namely, Lopinavir and Ritonavir tablets, Efavirenz tablets, and Tenofovir tablets, published in the International Pharmacopeia (4^th^ edition) by WHO. To assess the quality of medicines in this study, four different parameters including identity test, quantitative assay, uniformity of mass, and dissolution were selected for ARVs analysis. Counterfeit and substandard medicines are partly associated with the absence or insufficient quantities of the active substance. Therefore, the Identity test was conducted to confirm the presence and identity of the active substance in the tested formulation. The content and strength of drug were determined by a quantitative assay of the amount of active substance in dosage form. Uniformity of mass was tested to confirm homogeneity of the amount of active substances among tablets manufactured in the same batch. Dissolution of ARVs, with the exception of Efavirenz tablets due to the lack of an official analytical method in pharmacopeia, was conducted to test the performance of the ARV tablet that the drug substance will release with an acceptable rate which greatly affects the bioavailability of the medicine. Drug analysis was performed by the Pharmaceutical Technology Service Center, Faculty of Pharmaceutical Science, Chulalongkorn University. Pharmaceutical Technology Service Center is an accredited laboratory complying with the ISO/IEC 17025:2005 and the requirements of the Bureau of Laboratory Quality Standards in the field of drug testing. Evaluation of ARV quality was based on acceptance criteria of each specification described in the drug monograph, and descriptive summary statistics were calculated using Stata 14 (Statacorp, College Station, TX, USA). Sources and storage conditions were blinded from the analysts[16].

This research study was approved by the Institutional Review Board, Faculty of Medicine, Chulalongkorn University since October 2013. Informed consent process is not required by the ethics committee/IRB because the study was conducted and analyzed for the medicine tablets that were collected. This study was not conducted in patients.

This research study was not conducted in collaboration with Thailand Drug Regulation Authority (Thai FDA) because one objective of this study was to create an independent medicine quality surveillance system. However, a summary of the study results will be disseminated to relevant agencies and organizations with an interesting drug safety and quality.

## Results

Forty-two batches of ARV (TDF, EFV, LPV/r) from 15 sources (10 primary hospitals, 2 NGO clinics and 3 private drug stores) were collected between January–March 2015. Temperature during shipment of ARV samples from sites to the analysis facility did not substantially exceed 30 ˚C, except for 5 shipments from primary hospitals where the temperature was over 30^0^ C for ≤2 hours. All ARV samples came in the original package and no broken pills were observed. Of 15 TDF samples collected, 10 samples (from all hospitals) were made locally in Thailand (not WHO prequalified); the rest were generics made in India (3 WHO prequalified, 1 not) and Vietnam (1 not WHO prequalified). Fifteen samples of EFV were collected. Two samples (1 each from a primary hospital and 1 from an NGO clinic) were Thai-made generics (not WHO prequalified), 1 sample was a generic made in China (WHO prequalified) and the rest were Indian-made generics (WHO prequalified). LPV/r obtained from the 10 hospital sources was a Thai-made generic formulation (not WHO prequalified). We also sampled 1 Thai-made (not WHO prequalified) and 1 Indian-made (WHO prequalified) LPV/r generic tablets from 2 private sources (Assess the WHO prequalification database at http://apps.who.int/prequal/)[17,18] [Tables 1, 2 and 3].

**Table 1:** Lopinavir (200 mg)/Ritonavir (50 mg) drug sources and characteristics.

Site	Lot No.	Manufacturer	Country	Trade name	WHO Prequal.	Expiry date	Identification	% of label amount	Uniformity of mass, %		Dissolution, % (LPV/RTV)	
									Min	Max	Min	Max
001	W570041	GPO	THA	Lopinavir/Ritonavir	NO	21 Jan 16	Positive	102.1/100.8	-1.20	1.64	97.6/98.8	98.2/99.7
002	W560404	GPO	THA	Lopinavir/Ritonavir	NO	13 Jul 15	Positive	103.2/102.4	-1.28	1.48	98.1/100.8	99.1/102.2
003	W570169	GPO	THA	Lopinavir/Ritonavir	NO	17 Mar 16	Positive	102.1/103.6	-1.11	1.25	99.1/100.0	101.2/103.1
004	W570175	GPO	THA	Lopinavir/Ritonavir	NO	17 Mar 16	Positive	101.0/103.3	-2.22	2.08	97.7/99.0	101.9/102.5
005	W570450	GPO	THA	Lopinavir/Ritonavir	NO	17 Jul 16	Positive	99.4/101.8	-2.16	1.07	97.5/99.0	101.3/102.8
006	W560432	GPO	THA	Lopinavir/Ritonavir	NO	23 Jul 15	Positive	99.0/103.0	-2.10	1.87	98.5/100.3	102.6/104.6
007	W570457	GPO	THA	Lopinavir/Ritonavir	NO	20 Jul 16	Positive	98.6/101.5	-1.74	2.05	98.7/95.0	99.8/97.0
008	W570152	GPO	THA	Lopinavir/Ritonavir	NO	10 Mar 16	Positive	99.4/101.4	-2.09	1.76	96.2/99.0	98.7/102.8
009	W570285	GPO	THA	Lopinavir/Ritonavir	NO	15 May 16	Positive	98.4/101.0	-2.53	1.94	96.8/99.9	99.8/102.7
010	W560556	GPO	THA	Lopinavir/Ritonavir	NO	22 Oct 15	Positive	97.8/101.5	-1.47	1.70	99.1/101.4	101.7/103.7
011	W570169	GPO	THA	Lopinavir/Ritonavir	NO	17 Mar 16	Positive	98.2/101.2	-1.33	1.52	94.9/100.0	97.6/102.8
012	8000458	Mylan	IND	Lopinavir/Ritonavir	YES	30 Jun 16	Positive	99.7/101.4	-1.69	1.31	93.4/96.3	98.6/102.9

**Abbreviations:** Lopinavir,LPV; Ritonavir, RTV; the Government Pharmaceutical Organization, GPO; Thailand, THA; India, IND.

**Table 2:** Tenofovir (300 mg) drug sources and characteristics.

Site	Lot No.	Manufacturer	Country	Trade name	WHO Prequal.	Expiry date	Identification	% of label amount	Uniformity of mass, %		Dissolution, %	
									Min	Max	Min	Max
013	A570270	GPO	THA	Tenofovir GPO 300	NO	12 Feb 16	Positive	103.0	-0.81	1.46	102.3	103.3
014	A562265	GPO	THA	Tenofovir GPO 300	NO	27 Jun 15	Positive	100.0	-1.84	1.08	100.5	101.2
015	A570273	GPO	THA	Tenofovir GPO 300	NO	12 Feb 16	Positive	101.6	-1.44	1.26	101.1	102.8
016	A570554	GPO	THA	Tenofovir GPO 300	NO	18 Mar 16	Positive	99.6	-1.16	1.17	99.6	101.5
017	A570375	GPO	THA	Tenofovir GPO 300	NO	20 Feb 16	Positive	102.4	-1.34	0.70	98.7	101.8
018	A570324	GPO	THA	Tenofovir GPO 300	NO	17 Feb 16	Positive	103.4	-1.45	0.80	99.3	101.4
019	A570560	GPO	THA	Tenofovir GPO 300	NO	20 Mar 16	Positive	100.3	-1.42	1.19	100.9	102.4
020	A570322	GPO	THA	Tenofovir GPO 300	NO	13 Feb 16	Positive	98.6	-1.08	1.21	100.2	102.5
021	A570557	GPO	THA	Tenofovir GPO 300	NO	19 Mar 16	Positive	98.4	-0.79	1.19	101.0	104.3
022	A570742	GPO	THA	Tenofovir GPO 300	NO	22 Apr 16	Positive	97.0	-0.82	1.18	101.0	102.2
023	8027079	Mylan	IND	RICOVIR	YES	31 Jul 17	Positive	102.5	-4.54	4.47	98.2	100.9
024	020414	STADA	VN	Tenofovir STADA	NO	02 Feb 16	Positive	100.6	-2.49	2.05	96.9	100.9
025	2507153	RANBAXY	IND	TEVIR	NO	31 Mar 15	Positive	98.0	-1.42	1.21	100.3	101.6
026	8027079	Mylan	IND	RICOVIR	YES	31 Jul 17	Positive	98.4	-2.61	3.55	96.7	101.0
027	E131707	Hetero	IND	TENOF	YES	31 Jul 15	Positive	97.5	-2.10	2.14	90.3	109.0

**Abbreviations:** Tenofovir, TDF; the Government Pharmaceutical Organization, GPO; Thailand, THA; India, IND; Vietnam, VN.

**Table 3:** Efavirenz (600 mg) drug sources and characteristics.

Site	Lot No.	Manufacturer	Country	Trade name	WHO Prequal.	Expiry date	Identification	% of label amount	Uniformity of mass, %		Dissolution, %	
									Min	Max	Min	Max
028	EM27186	Mylan	IND	Efavirenz	YES	31 May 17	Positive	96.3	-2.22	1.25	-	-
029	EM35108	Emcure	IND	Efavirenz	YES	30 Apr 15	Positive	96.8	-1.83	2.21	-	-
030	3027189	Mylan	IND	Efavirenz	YES	31 May 17	Positive	96.9	-1.86	1.62	-	-
031	3027187	Mylan	IND	Efavirenz	YES	31 May 17	Positive	97.6	-2.85	1.67	-	-
032	3027185	Mylan	IND	Efavirenz	YES	31 May 17	Positive	97.3	-2.21	1.84	-	-
033	3027486	Mylan	IND	Efavirenz	YES	31 May 17	Positive	97.8	-1.06	0.80	-	-
034	3031217	Mylan	IND	Efavirenz	YES	30 Sep 17	Positive	97.6	-1.11	1.14	-	-
035	A570303	GPO	THA	Efavirenz	NO	10 Sep 15	Positive	95.4	-1.56	1.28	-	-
036	3031198	Mylan	IND	Efavirenz	YES	31 Aug 17	Positive	97.8	-1.51	1.83	-	-
037	3031240	Mylan	IND	Efavirenz	YES	30 Sep 17	Positive	97.7	-1.76	1.91	-	-
038	3026355	Mylan	IND	EFAMAT	YES	30 Apr 17	Positive	96.8	-2.25	2.08	-	-
039	EFZ113033A	Hetero	IND	ESTIVA-600	YES	30 Nov 16	Positive	98.9	-0.98	0.86	-	-
040	A570845	GPO	THA	Efavirenz	NO	22 Oct 15	Positive	95.6	-2.08	2.25	-	-
041	Y1723	Zhejian Huahai Pharm.	CHI	STOCRIN	YES	03 Mar 16	Positive	95.6	-1.01	0.75	-	-
042	E140542	Hetero	IND	ESTIVA-600	YES	28 Feb 17	Positive	94.9	-2.59	1.02	-	-

**Abbreviations:** Efavirenz, EFV; the Government Pharmaceutical Organization, GPO; Thailand, THA; China, CHI; India, IND.

For ARV obtained from hospital sources, *ARV Storage conditions* were assessed from self-administered questionnaires completed by a hospital pharmacist. Nine out of 10 hospitals had air conditioned storage facilities with temperature less than 30°C. Seven out of 10 hospitals had humidity monitoring. Humidity was less than 60% relative humidity (RH) at most of the hospitals where monitoring was undertaken. There was 1 site with humidity of 67%RH. One hospital site had neither humidity nor temperature monitoring, because drugs were stored at the ARV clinic, not in pharmacy facility. NGO ARV clinics also had temperature and humidity controlled to be less than 30°C and 60%RH respectively. We could not assess storage conditions at private pharmacies. Of note, while a doctor’s prescription is required for ARV dispensed at hospitals and the NGO ARV clinics, no formal prescription was needed in the private pharmacies where our ARV samples were procured.

### Identification test and Chemical composition assay

**Fig 3:**
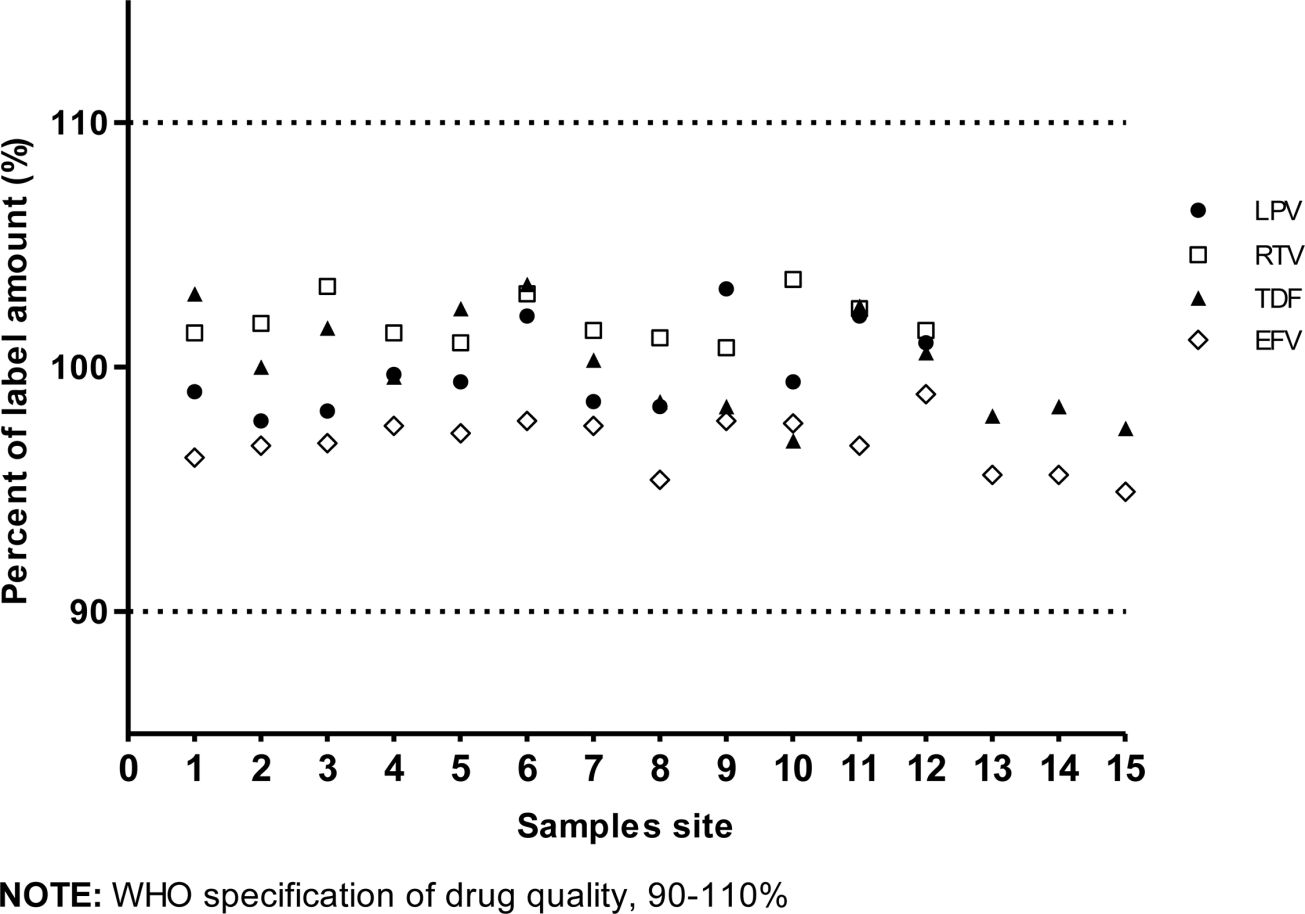
Percent of label amount of each sampled ARV, by sampling site.

The drug identities are demonstrated by the same retention time as corresponding International pharmacopeia reference standards using the relevant drug content assay. Each sample met the International pharmacopeia standard for drug content [Fig 3]. Mean drug content values, as described in [Table 4], were close to 100% of the labelled amount; 99.9%/101.9% for LPV/r, 100% for TDF, and 96.9% for EFV. The standard deviations of all drug content assay values were relatively close; 1.76/0.93 for LPV/r, 2.10 for TDF, and 1.11 for EFV.

**Table 4:** Descriptive statistics of drug content, uniformity of mass and dissolution tests.

ARV	N	% of label amount (L.A.)				Uniformity of mass, %			Dissolution, %		
		WHO Specification	Min	Max	Mean (SD)	WHO Spec.	Min	Max	WHO Spec.	Min	Max
LPV/RTV	12	90.0–110.0%	97.8/100.8	103.2/103.6	99.9 (1.76)/101.9 (0.93)	±5%	-2.53	2.08	≥ 80% L.A.	93.4/95.0	102.6/104.6
TDF	15	90.0–110.0%	97.0	103.4	100.1 (2.10)	±5%	-4.54	4.47	≥ 80% L.A.	90.3	109.0
EFV	15	90.0–110.0%	94.9	98.9	96.9 (1.11)	±5%	-2.85	2.25	-	-	-

**Abbreviations:** Lopinavir,LPV; Ritonavir, RTV; Tenofovir, TDF; Efavirenz, EFV; Label amount, L.A.; Standard deviation, SD

### Uniformity of mass

Uniformity of mass was used to assess uniformity of production batch for all the samples. The results ranged from -2.53% to 2.08% for LPV/r, -4.54% to 4.47% for TDF and -2.85% to 2.25% for EFV. All results were within the accepted values which range from -5% to 5%. The % deviation under uniformity of mass is calculated from the difference between the weight of individual unit and the average weight of the sample. The acceptance range is based on the criteria in the International pharmacopeia.

### Test for dissolution

For dissolution tests, the cumulative amount of drug (percentage of labelled amount) that dissolves over a period of time in a dissolution medium is measured. As shown in Table 4, LPV/r and TDF samples comply with the international pharmacopeia dissolution test limit of ≥ 80% of the labeled amount.

There was no significant association between sources of ARV, WHO prequalification status, manufactured sites and storage conditions and the results of this pharmaceutical equivalence analysis.

## Discussion

The primary goal of this research study was to perform independent surveillance on the quality of commonly used generic ARV available for patients in Thailand. Although this could not represent whole ARV distributing in the region, the findings showed satisfactory quality of all ARV samples from different sources and types based on drug content, uniformity of mass and dissolution even though some batches (including those manufactured in Thailand) are not WHO prequalified. TDF test results varied most widely when compared to EFV and LPV/r, however all parameters were within the International Pharmacopeia standards. These wider ranges might reflect more variability in manufacturing sites of TDF, while LPV/r samples were retrieved from a smaller number of manufacturers. Although our findings are supported by previous studies[19–21], two samples of ARV from Thailand were found to be substandard in a USP convention database report in 2008 (http://www.usp.org/worldwide/medQualityDatabase) [22]. Therefore, continuous monitoring is required to ensure that products used in National Treatment Programs meet the quality standards necessary to ensure an effective and safe response to HIV. In addition, counterfeit medicines, particularly anti-malarials have frequently been found when reviewing the quality of medicines in Southeast Asian countries [11,12], and WHO estimates the use of counterfeit drugs causes approximately 1 million deaths per year[23].

Although ARVs are life-saving medications, they can cause adverse events if taken incorrectly and reduce a patient’s options for future treatment if they continue to be dosed in the presence of genotypic resistance. HIV care is life-long and requires multidisciplinary support, and monitoring of viral load and other safety parameters. Our finding that generic ARV could be purchased from two private pharmacies without a formal prescription, despite their supply being restricted only to hospitals and registered clinics, highlights the important public health issue of getting patients into the proper healthcare system cascade. Self-treatment with ARV, either for treatment or prevention, without proper follow up from healthcare professionals skilled in managing HIV might lead to adverse events, adherence problems, and consequently drug resistance[24–26]. While we promote ARV accessibility by generic importation/licensing, regulation is necessary to ensure that ARV are dispensed with a proper monitoring and follow-up system in place.

There are some limitations of our study. First, the small number of ARV samples assayed limits the power to detect any abnormalities. However, we maximized our resources by sampling ARV from primary hospitals which in theory, have the least rigorous quality control systems in place. The fact that hospital ARV samples were selected by local pharmacists, could potentially result in a “positive” selection bias Second, since there is no mechanism to check the availability of ARV in facilities outside the NHSO system, convenience sampling was used for ARV from private pharmacies and NGO’s in locations where we suspected ARV would be available. This approach could miss stores in other areas who might have substandard products. Third, almost all LPV/r tablet sampled were Thai-made generics; this reflects the situation in the past when Thailand issued compulsory licenses for antiretroviral drugs including Abbott’s Kaletra®, to cope with the enormous expenditure incurred by procurement of brand name ARV. Nevertheless, although Thai ARV are not WHO pre-qualified, a study has shown comparable pharmacokinetics between Thai generic and Indian generics [27], and the Thai FDA has rigorous standards for bioequivalence[8]. Finally, we were unable to include all pharmaceutical analysis methods, namely impurities and related substances, thus not allowing to us to exclude the presence of possible contaminants and resulting potential for toxicity. However, we covered the three most important components relating to bioavailability and efficacy: active ingredient, uniformity and dissolution. Some strengths of our study are also noteworthy. The collection of ARV were processed in systematic fashion. In addition, our ARV sources and storage conditions were blinded from analysts so they did not pose any bias during analysis. Lastly, while occasional surveillance of drug quality in Thailand already takes place [28], the surveillance might not focus on ARV. Our study is independent and focuses on commonly used ARV in the Thai National Treatment Program.

In conclusion, many sectors in Thailand have worked together to scale up ARV treatment; this study ensures the quality of the sampled drug being utilized, emphasizes further continuous monitoring, and at the same time, points out that ARV dispensing should occur in facility-based settings where regular follow-up and care are delivered.

## Supporting Information

S1 Data Minimum data set. (XLS)

S1 Table Descriptive statistics of Lopinavir (200 mg)/Ritonavir (50 mg) drug content, uniformity of mass and dissolution tests, by WHO pre-qualification status, Sampling site, and country of manufacture. (DOCX)

S2 Table Descriptive statistics of Tenofovir drug (300 mg) content, uniformity of mass and dissolution tests, by WHO pre-qualification status, Sampling site, and country of manufacture. (DOCX)

S3 Table Descriptive statistics of Efavirenz drug (600 mg) content, uniformity of mass and dissolution tests, by WHO pre-qualification status, Sampling site, and country of manufacture. (DOCX)
