# Supplementary material for: Pharmaceutical Equivalence of Distributed Generic Antiretroviral (ARV) in Asian Settings: The Cross-Sectional Surveillance Study – PEDA Study
Source: PLoS One. 2016 Jun 20;11(6):e0157039. doi: 10.1371/journal.pone.0157039 (PMC4913952; doi:10.1371/journal.pone.0157039)
Supplement: S1 Table — (DOCX) [file pone.0157039.s002.docx]

**S1 Table. Descriptive statistics of Lopinavir (200 mg)/Ritonavir (50 mg) drug content, uniformity of mass and dissolution tests, by WHO pre-qualification status, Sampling site, and country of manufacture**

| Variables | **N of 12** | **% of label amount (L.A.) (WHO Specification: 90.0-110.0%)** | | |  | **Uniformity of mass, % (WHO Spec. ±5%)** | |  | **Dissolution, % (WHO Spec. ≥ 80% L.A.)** | |
| --- | --- | --- | --- | --- | --- | --- | --- | --- | --- | --- |
|  |  | **Min** | **Max** | **Mean (SD)** |  | **Min** | **Max** |  | **Min** | **Max** |
| **WHO pre-qualification status** |  |  |  |  |  |  |  |  |  |  |
| Yes | 1 | - | - | 99.7 (0)/101.4 (0) |  | -1.69 | 1.31 |  | 93.4/96.3 | 98.6/102.9 |
| No | 11 | 97.8/100.8 | 103.2/103.6 | 99.9 (1.85)/102.0 (0.97) |  | -2.53 | 2.08 |  | 94.9/95.0 | 102.6/104.6 |
| **Sampling sites** |  |  |  |  |  |  |  |  |  |  |
| Hospital | 10 | 97.8/100.8 | 103.2/103.6 | 100.1 (1.86)/102.0 (0.99) |  | -2.53 | 2.08 |  | 96.2/95.0 | 102.6/104.6 |
| NGO Clinic | - | - | - | - |  | - | - |  | - | - |
| Private | 2 | 98.2/101.2 | 99.7/101.4 | 99.0 (1.06)/101.3 (0.14) |  | -1.69 | 1.52 |  | 93.4/96.3 | 98.6/102.9 |
| **Manufacturer Countries** |  |  |  |  |  |  |  |  |  |  |
| Thailand | 11 | 97.8/100.8 | 103.2/103.6 | 99.9 (1.85)/102.0 (0.97) |  | -2.53 | 2.08 |  | 94.9/95.0 | 102.6/104.6 |
| India | 1 | - | - | 99.7 (0)/101.4 (0) |  | -1.69 | 1.31 |  | 93.4/96.3 | 98.6/102.9 |

**Abbreviations:** Non-Governmental Organizations, NGO
