# Supplementary material for: Pharmaceutical Equivalence of Distributed Generic Antiretroviral (ARV) in Asian Settings: The Cross-Sectional Surveillance Study – PEDA Study
Source: PLoS One. 2016 Jun 20;11(6):e0157039. doi: 10.1371/journal.pone.0157039 (PMC4913952; doi:10.1371/journal.pone.0157039)
Supplement: S2 Table — (DOCX) [file pone.0157039.s003.docx]

**S2 Table. Descriptive statistics of Tenofovir drug (300 mg) content, uniformity of mass and dissolution tests, by WHO pre-qualification status, Sampling site, and country of manufacture**

|  |  | **% of label amount (L.A.) (WHO Specification: 90.0-110.0%)** | | |  | **Uniformity of mass, % (WHO Spec. ±5%)** | |  | **Dissolution, % (WHO Spec. ≥ 80% L.A.)** | |
| --- | --- | --- | --- | --- | --- | --- | --- | --- | --- | --- |
|  | N of 15 | Min | Max | Mean (SD) |  | Min | Max |  | Min | Max |
| **WHO pre-qualification Status** |  |  |  |  |  |  |  |  |  |  |
| Yes | 3 | 97.5 | 102.5 | 99.5 (2.67) |  | -4.54 | 4.47 |  | 90.3 | 109 |
| No | 12 | 97 | 103.4 | 100.2 (2.05) |  | -2.49 | 2.05 |  | 96.9 | 104.3 |
| **Sampling sites** |  |  |  |  |  |  |  |  |  |  |
| Hospital | 10 | 97 | 103.4 | 100.4 (2.13) |  | -1.84 | 1.46 |  | 98.7 | 104.3 |
| NGO Clinic | 2 | 97.5 | 98 | 97.8 (0.35) |  | -2.1 | 2.14 |  | 90.3 | 109 |
| Private | 3 | 98.4 | 102.5 | 100.5 (20.5) |  | -4.54 | 4.47 |  | 96.7 | 101 |
| **Manufacturer Countries** |  |  |  |  |  |  |  |  |  |  |
| Thailand | 10 | 97 | 103.4 | 100.4 (2.12) |  | -1.84 | 1.46 |  | 98.7 | 104.3 |
| India | 4 | 97.5 | 102.5 | 99.1 (2.30) |  | -4.54 | 4.47 |  | 90.3 | 109 |
| Vietnam | 1 | - | - | 100.6 (0) |  | -2.49 | 2.05 |  | 96.9 | 100.9 |

**Abbreviations:** Non-Governmental Organizations, NGO
