# Supplementary material for: Pharmaceutical Equivalence of Distributed Generic Antiretroviral (ARV) in Asian Settings: The Cross-Sectional Surveillance Study – PEDA Study
Source: PLoS One. 2016 Jun 20;11(6):e0157039. doi: 10.1371/journal.pone.0157039 (PMC4913952; doi:10.1371/journal.pone.0157039)
Supplement: S3 Table — (DOCX) [file pone.0157039.s004.docx]

**S3 Table. Descriptive statistics of Efavirenz drug (600 mg) content, uniformity of mass and dissolution tests, by WHO pre-qualification status, Sampling site, and country of manufacture**

|  | N of 15 | **% of label amount (L.A.) (WHO Specification: 90.0-110.0%)** | | |  | **Uniformity of mass, % (WHO Spec. ±5%)** | |  | **Dissolution, % (WHO Spec. ≥ 80% L.A.)** | |
| --- | --- | --- | --- | --- | --- | --- | --- | --- | --- | --- |
|  |  | **Min** | **Max** | **Mean (SD)** |  | **Min** | **Max** |  | **Min** | **Max** |
| **WHO pre-qualification status** |  |  |  |  |  |  |  |  |  |  |
| Yes | 13 | 94.9 | 98.9 | 97.1 (1.04) |  | -2.85 | 2.21 |  | - | - |
| No | 2 | 95.4 | 95.6 | 95.5 (0.14) |  | -2.08 | 2.25 |  | - | - |
| **Sampling sites** |  |  |  |  |  |  |  |  |  |  |
| Hospital | 10 | 95.4 | 97.8 | 97.1 (0.78) |  | -2.85 | 2.21 |  | - | - |
| NGO Clinic | 2 | 94.9 | 95.6 | 95.3 (0.49) |  | -2.59 | 2.25 |  | - | - |
| Private | 3 | 95.6 | 98.9 | 97.1 (1.67) |  | -2.25 | 2.08 |  | - | - |
| **Manufacturer Countries** |  |  |  |  |  |  |  |  |  |  |
| Thailand | 2 | 95.4 | 95.6 | 95.5 (0.14) |  | -2.08 | 2.25 |  | - | - |
| India | 12 | 94.9 | 98.9 | 97.2 (0.99) |  | -2.85 | 2.21 |  | - | - |
| China | 1 | - | - | 95.6 (0) |  | -1.01 | 0.75 |  | - | - |

**Abbreviations:** Non-Governmental Organizations, NGO
